# Machine learning for classification of periodontal defects (vertical versus horizontal) using CBCT datasets

**DOI:** 10.6026/973206300220953

**Published:** 2026-02-28

**Authors:** Sourav Panda, Vishwannath Hiremath, J Sophia Jeba Priya, Nimisha Sanjay Pagare, Rachita Mustilwar, Sunny Mavi

**Affiliations:** 1 Department of Periodontics, Institute of Dental Sciences, Siksha O Anusandhan University, Bhubaneswar, India; 2 Department of Oral and Maxillofacial Surgery, A Unit of Hiremath Hospitals Pvt Ltd, Vijayanagar, Bangalore, India; 3 Department of Oral Medicine and Radiology, Tamilnadu Government Dental College and hospital, Chennai, India; 4 Department of Periodontology, Dr. D.Y. Patil Dental College and Hospital, Dr. D.Y. Patil Vidyapeeth (Deemed to be University), Pune, Maharashtra, India; 5 Department of Periodontology, Rural Dental College, Pravara Institute of Medical Sciences, Maharashtra, India; 6 Department of Periodontics, Sudha Rustagi College of Dental Sciences and Research, Faridabad, Haryana, India

**Keywords:** Machine learning, periodontal defects, cone-beam computed tomography, deep learning, classification, artificial intelligence

## Abstract

Accurate classification of periodontal bone defects is essential for treatment planning; yet conventional radiographic methods have limitations in complex anatomical regions. Therefore, it is of interest to develop and validated machine learning models to automatically classify periodontal bone defects using cone-beam computed tomography data from 1,847 teeth in 312 patients treated between January 2021 and December 2023. Convolutional neural networks, random forest, support vector machines and gradient boosting classifiers were trained using radiomic features and raw image data, with performance evaluated through five-fold cross-validation against expert consensus. The convolutional neural network achieved the highest performance with 91.4% accuracy, 89.8% sensitivity for vertical defects, 92.6% specificity for horizontal defects and an area under the ROC curve of 0.946. Thus, we show that machine learning; particularly deep learning approaches can reliably classify periodontal defect morphology on CBCT images and support improved diagnostic consistency and clinical decision-making in periodontology.

## Background:

Periodontal disease is a major burden in oral health on the world, with an estimated 50 percent of adult populations worldwide suffering the disease as the primary cause of tooth loss in adults [1]. The tissue-pathological alteration of the periodontal supporting structures, such as the alveolar bone, periodontal ligament and cementum, occurs in distinctive patterns which are the basis of determining treatment strategies and prognostic evaluation [2]. The division of alveolar bone defects into vertical (angular) and horizontal shapes have great clinical implications, with defect morphology having a direct effect on treatment choice, regenerative capabilities and prognosis [3]. Vertical defect, where there is loss of bone at the root surface in an angular manner with the bony walls remaining, provides good circumstances to revitalizing operations such as guided tissue regeneration and bone grafting. On the other hand, horizontal defects, which imply a consistent crestal bone loss over the affected regions, normally necessitate other management measures [4]. Conventional radiographic evaluation of periodontal bone defects depends mainly on two- dimensional intraoral periapical radiographs and panoramic radiographs, which do not give much information about the buccal-lingual dimensions and the three-dimensional architecture of defects [5]. The diagnostic accuracy of traditional radiographic methods is further undermined by superimposition of anatomical structures, geometric distortion and positioning that depends on the operator [6]. Cone-beam computed tomography has now become a useful imaging technique in periodontal evaluation and has provided a three-dimensional view of alveolar bone morphology with a significant lower dose of radiation than traditional computed tomography [7].

CBCT facilitates accurate assessment of defect size, wall structures and space relationships which cannot be observed through the traditional radiography [8]. Nonetheless, CBCT datasets have to be interpreted with specialized knowledge and specific time investments which imposes a limit to the number of practices that can be adopted within a normal periodontal practice [9]. The use of artificial intelligence in dental imaging has spread at an unprecedented rate and the machine learning algorithms prove to be very efficient in automated detection and classification tasks in various diagnostic fields [10]. The performance of deep learning methods, especially convolutional neural networks, has been found to be on par or better than expert clinicians in a number of image-based diagnostic tasks in dentistry [11]. The use of machine learning applications directly related to periodontal disorders has demonstrated prospective outcomes in performing and fulfilling the following activities: detecting periodontitis, measuring bone loss and staging [12]. Potential of automated periodontal examination has been proven by the use of panoramic radiographs but there are limited studies assessing the use of three-dimensional CBCT-based methodologies [13]. Automated classification of periodontal defect morphology with the help of machine learning provides a notable clinical requirement and can increase diagnostic consistency, decrease the time required to interpret the classification results and evidence-based treatment planning [14]. Radiomic feature reconstruction of CBCT images provides the ability to describe defect patterns quantitatively which might be able to capture fine morphological differences not observable by human eyes [15]. Although there is a recent surge in the use of artificial intelligence to solve periodontal imaging tasks, a comprehensive exploration of machine learning methods to classify vertical and horizontal defects using CBCT has not been done [16]. Therefore, it is of interest to describe the development and validation of machine learning-based models for automated classification of periodontal bone defects into vertical and horizontal patterns using CBCT data.

## Materials and Methods:

## Study design and setting:

It was a retrospective diagnostic accuracy study that was carried out at the Department of Periodontology and Oral Radiology, University Dental Hospital, in the period between January 2021 and December 2023. The research made use of archived CBCT scans of the patients who had undergone imaging to have periodontal evaluation and treatment planning purposes.

## Sample selection:

CBCT scans had been accessed in the institutional imaging database along the following lines:

## Inclusion criteria:

[1] Adult patients who are 18 years and over.

[2] CBCT scans protocol obtained in a standard institutional protocol.

[3] Presence of periodontal bone loss as evidenced by at least one tooth in the radiograph.

[4] Adequate image quality to be used in the evaluation of defects.

[5] Full visualization of teeth and alveolar structures.

## Exclusion criteria:

[1] Existence of periapical pathology in assessment.

[2] Endodontic- Periodontal lesions.

[3] Teeth that have a large restoration and result in severe artifacts.

[4] Third molars.

[5] Root-resorptive or developmentally-abnormal teeth.

[6] Motion artifacts or unfinished field of view scans.

[7] Had evidence of regenerative periodontal procedures done in the past.

## CBCT acquisition protocol:

A standardized protocol on Carestream CS 9300 (Carestream Dental LLC, Atlanta, GA, USA) or Planmeca ProMax 3D (Planmeca Oy, Helsinki, Finland) was used to acquire all CBCT scans. The parameters of the acquisition were: field-of-view 8x8 cm or 10x10 cm (region -specific), voxel size was 0.15-0.20 mm, tube voltage 84-90 kV, tube current 4-8 mA and exposure time 12-20 seconds. Exportation of DICOM datasets was done to be processed and analyzed.

## Reference standard classification:

The reference standard that was used to train models and validate them was expert consensus classification. Each tooth with bone loss was examined independently in three independently evaluated tooth positions (three board certified periodontists with at least 10 years of clinical experience).

[1] Vertical (Angular) defect: Oblique or angular bone loss along the root surface, the base of the defect will be apical to the adjacent crestal bone level and has at least one remaining bony wall.

[2] Horizontal defect: Compared to the bottom, the same reduction of marginal bone height, which is relatively parallel to the cementoenamel junction, with no angular constituent or residual walls.

[3] Combined/Complex: Malformations with the properties of both patterns.

In this research, the combined defects were not considered to allow binary classification. Cohen kappa was used to determine initial inter-examiner agreement on all inter-examiner cases and discordant cases were resolved by consensus. The final classification needed approvals of two examiners or unanimous decision after discussion.

The 3D-CNN model significantly outperformed all traditional machine learning models (McNemar's test, p < 0.01 for all pairwise comparisons). Among traditional models, Random Forest achieved the highest performance, significantly exceeding logistic regression (p < 0.001) and KNN (p < 0.001). Model performance varied across tooth types and defect characteristics. Classification accuracy was highest for premolars (93.2%) and lowest for molars (88.6%), potentially reflecting greater anatomical complexity in molar regions (Table 3). Analysis of misclassified cases revealed that errors predominantly occurred in shallow defects (< 3mm) and cases with transitional morphology between defect types. Molars with furcation involvement demonstrated higher misclassification rates. For the Random Forest model, feature importance analysis identified shape-based features as most discriminative, with elongation, flatness and sphericity ranking highest. Among texture features, gray-level run length non-uniformity and gray-level co-occurrence matrix correlation contributed substantially to classification. Gradient-weighted class activation mapping (Grad-CAM) visualization for the 3D-CNN model demonstrated appropriate attention focus on defect margins and bone-root interfaces, confirming that the model learned clinically relevant anatomical features for classification decisions. Five-fold cross-validation demonstrated consistent performance across folds for the 3D-CNN model, with mean accuracy of 90.8 ± 1.4% and AUC-ROC of 0.942 ± 0.012, indicating robust generalization without significant over fitting.

## Image processing and segmentation:

Pre-processing of CBCT datasets was done with 3D Slicer program (version 5.0, www.slicer.org) to have standardized pre-processing.

## The following steps were introduced:

[1] Reorientation: Datasets were resorted to standardized anatomical planes having occlusal plane horizontal.

[2] Region of interest extraction: Single tooth regions were separated using standardized margins (2mm above a root apex and 3mm peripheral to root surfaces).

[3] Intensity matching: Intensity matching between histograms of scans of different machines was used.

[4] Noise reduction: Non-local means denoising filter using sigma = 0.05.

[5] Resampling: Resampling of all volumes to isotropic voxel size of 0.2mm.

## Feature extraction:

Two parallel model input preparation methods were used:

## Approach A - radiomic feature extraction:

The 107 quantitative features were extracted in each segmented defect region by pyRadiomics library (version 3.0) and they included:

[1] First order statistics (18 features): Mean variance, skewness, kurtosis, energy, entropy, etc.

[2] Features related to shape (14 features): volume, surface area, sphericity, elongation, flatness, etc.

[3] Features of the gray-level co-occurrence matrix (24 features): contrast, correlation, homogeneity, energy, etc.

[4] Gray-level run length matrix characteristics (16 characteristics).

[5] Features of gray-level size zone matrices (16 features).

[6] Features of neighboring gray tone difference matrices (5 features).

[7] Gray-level dependence matrix features (14 features).

The feature selection was performed by the use of recursive feature elimination with cross-validation in order to select the best feature subsets.

## Approach B - raw image input:

Ready-to-input deep learning models were created with standardized 3D volumes (64x64x64 voxels) that were centered on the defect area. Random rotation (±15), scaling (0.9-1.1) and intensity variation (±10 percent) were used in data augmentation during training.

## Machine learning models:

## Several machine learning algorithms were applied and compared:

[1] Radiomomic features are used to create traditional Machine Learning Models:

[2] Random Forest (RF): Collection of 500 decision trees whose maximum depth is 20 and minimum samples split is 5.

[3] Support Vector machine (SVM): Radial basis kernel and hyper parameter optimization through grid search.

[4] Gradient Boosting Classifier (GBC): 200 estimators, learning rate 0.1 and maximum depth 6.

[5] Logistic Regression (LR): L2 regularization and the hyperparameter optimization.

[6] K-Nearest Neighbors (KNN): The best parameter was identified by cross-validation.

[7] Deep Learning Models (raw image based):

[8] 3D Convolutional Neural Network (3D-CNN): Custom architecture 4 convolutional blocks (32, 64, 128, 256 filters), batch normalization, ReLU activation, max pooling and dense layers (512, 256, 128 units) with a dropout (0.5).

[9] ResNet-18 3D: Adaptation of residual network architecture to 3D volumetric input.

[10]DenseNet-121 3D: 3D convolution dense connection architecture.

[11]PyTorch (version 1.12) was used to implement deep learning models and was trained on NVIDIA RTX 3090 GPU. The following were training parameters: learning rate = 0.0001 (Adam optimizer), early stopping at patience = 20 epochs and maximum 200 epochs.

## Model training and testing:

The data were randomly divided into training (70 percent), validation (15 percent) and test (15 percent) sets where stratification was done to preserve density. The combined training and validation sets were hyperparameter tuned and the performance estimated by cross- validating the combined sets 5 times. The model was assessed using the held-out test set. To solve the issue of class imbalance, weighted loss functions and oversampling of the minority class with SMOTE (Synthetic Minority Over-sampling Technique) were used in conventional ML models.

## Performance metrics:

## Model performance was evaluated using:

[1] Accuracy: (TP + TN) / (TP + TN + FP + FN)

[2] Sensitivity (Recall): TP / (TP + FN)

[3] Specificity: TN / (TN + FP)

[4] Precision: TP / (TP + FP)

[5] F1-Score: 2 x (Precision x Recall) / (Precision + Recall)

[6] Area Under ROC Curve (AUC-ROC)

[7] Area Under Precision-Recall Curve (AUC-PR)

Confidence intervals (95%) were calculated using bootstrap resampling (1000 iterations). McNemar's test compared paired model predictions. Statistical significance was defined as p < 0.

## Results:

From 428 CBCT scans reviewed, 312 met inclusion criteria, yielding 2,156 teeth with periodontal bone loss. Following exclusion of 309 teeth with combined defects or other exclusion factors, 1,847 teeth comprised the final analytical dataset. The sample included 1,124 vertical defects (60.9%) and 723 horizontal defects (39.1%) (Table 1). Patient demographics showed mean age of 52.4 ± 11.8 years (range 24-78), with 168 males (53.8%) and 144 females (46.2%). Tooth distribution included 486 incisors (26.3%), 312 canines (16.9%), 428 premolars (23.2%) and 621 molars (33.6%). Inter-examiner agreement for initial classification was substantial (κ = 0.78, 95% CI: 0.74-0.82), with consensus achieved for all cases following discussion (Table 2). From 107 extracted radiomic features, recursive feature elimination identified 24 optimal features for classification. The most discriminative features included shape-based metrics (sphericity, flatness, elongation), first-order statistics (entropy, skewness, kurtosis) and texture features from gray-level co-occurrence and run-length matrices. Significant differences between defect types were observed for multiple features. Vertical defects demonstrated higher elongation values (0.68 ± 0.14 versus 0.42 ± 0.12, p < 0.001), lower sphericity (0.34 ± 0.11 versus 0.52 ± 0.13, p < 0.001) and distinct texture patterns characterized by higher gray-level run length non-uniformity. All machine learning models achieved classification performance exceeding baseline, with substantial variation across algorithms. The 3D-CNN model demonstrated superior overall performance with accuracy of 91.4% on the test set.

## Discussion:

In this paper, the paper illustrates successful implementation of machine learning to automated detection of periodontal defects based on CBCT imaging with deep learning structures performing very well in diagnostic tasks. The 3D-CNN model achieved an accuracy of more than 91% and AUC-ROC of 0.946, which are clinically relevant levels of classification that may be applied to improve the diagnostic reliability of periodontal practice. The high output of deep learning models relative to classic machine learning algorithms is consistent with a number of studies conducted in the field of medical imaging classification [17]. Convolutional neural networks are good at capturing hierarchical spatial features directly on image data that may include patterns that are not directly identified by predetermined radiomic features [18]. The end-to-end learning paradigm removes the possible loss of information in the process of manually engineered features. Radiomic feature-based method of the Radiomic classifier the Random Forest and Gradient Boosting however achieved an impressive result of above 85 percent accuracy, indicating that quantitative texture and shape characteristics harbor helpful discriminative data [19]. The implication of this finding is practical because the traditional machine learning models are less computationally demanding and could be easier to interpret by clinical users [20]. Shape-related characteristics, especially elongation and sphericity, became very discriminative and demonstrated the basic morphological difference between angular horizontal bone loss patterns and vertical defects [21]. The results are consistent with the clinical knowledge of defect morphology and the findings substantiate the radiomic method of periodontal imaging application. The noted difference in performance among the types of teeth, whereby the accuracy is lower in molars, probably relates to the higher anatomy complexity of multi-rooted-teeth, involvement of furcations and possibility of imaging artifacts [22]. Multi- rooted teeth offer three dimensional defect configurations that could have overlapping features between defect categories. Molar-region specialized architecture or training is an area that should be considered in the development of future models. The correlation that exists between the depth of defects and the accuracy of classification whereby moderate depths of defects (4-6mm) performed best implies that very shallow defects might not have the morphological difference that would enable them to be classified reliably [23]. This finding has clinical implications and both human interpreters and automated systems may have some difficulties with the diagnosis of defects at an early stage when classification is necessary to plan treatment. Analysis of wall configuration of vertical defects showed that with more walls the defect was classified better as was expected with well-defined three-wall defects having more characteristic angular morphology [24].

The lesions with the least clear-cut configuration had one-wall defects which proved the most misclassified, as it is expected that such lesions are difficult to assess clinically. The analysis of model interpretability by Grad-CAM visualization has established that the deep learning model concentrated on anatomically significant areas to make classification choices [25]. Concentration of attention at defect margins and bone-root interface implies that clinically meaningful features are learned and not spurious relationships and gives confidence to model validity. Comparisons with the existing literature in the AI of periodontal studies show that there are similarities in the applicability of automated evaluation of defects and that such direct comparison are hampered by differences in methodologies [26]. A number of studies which have used panoramic radiographs to detect periodontal bone loss have reported an accuracy of between 80-95 percent; however, three dimensional classifications of defects would be a unique and equally more difficult task [27]. Such classification systems might also offer clinical decision support related to the treatment planning process; however, it might be especially beneficial in clinical settings with less expert periodontal clinicians or in a clinical context that does not have expert periodontal knowledge [28]. The defect classification of pre-screening CBCT datasets automation could also facilitate the workflow and provide uniform documentation of defect patterns. There are a number of restrictions that should be mentioned. The single-institution retrospective design can also have poor generalizability to other groups of patients and imaging equipment. The combined defects were excluded which made classification easier but might not reflect clinical complexity where mixed patterns are frequent [29]. Although the standard of reference is presupposed by expert opinion, it is still subject to subjectivity in the classification of defects. The vertical defects in the model were predisposed by the class imbalance and necessitated methodological adjustments, which could affect the model calibration. External validation on independent datasets of other institutions and imaging systems is necessary prior to clinical implementation [30]. Also, the computational needs of deep learning models might be a challenge to implementation in resource constrained environments. The future research directions are expanded to multi-class classification based on combined defects and unique wall configurations, combining clinical and radiographic information to provide better predictions and creating fully automated pipelines that will include defect identification before the classification [31]. The future validation research on clinical impact of treatment planning decisions would be crucial evidence to implement it [32]. The opportunities of artificial intelligence to improve periodontal image interpretation are great and automated classification is one of the aspects of a full diagnostic support system [33]. End-to-end decisions on the regenerative therapy selection and prognosis estimation could be supported by the integration with treatment planning algorithms.

## Conclusion:

Random Forest and Gradient Boosting classifiers Radiomic feature based classification with a clinical useful accuracy of over 85 percent was also obtained using radiomic features, providing an alternative to deep learning with computational efficiency. Such features as the shape such as elongation and sphericity were found to be highly discriminative and they showed underlying morphological differences in defects. The differences in the performance of the types of teeth and defects demonstrate the significance of the overall assessment of the model and indicate possibilities of its specific enhancement in problematic areas of the anatomy.

## Figures and Tables

**Table 1:** Dataset characteristics and distribution of periodontal defects

**Characteristic**	**Vertical Defects (n=1,124)**	**Horizontal Defects (n=723)**	**Total (n=1,847)**	**p-value**
Patient Age (years)				
Mean ± SD	51.8 ± 11.6	53.4 ± 12.1	52.4 ± 11.8	0.084
Range	24-76	28-78	24-78	
Sex, n (%)				0.342
Male	612 (54.4)	378 (52.3)	990 (53.6)	
Female	512 (45.6)	345 (47.7)	857 (46.4)	
Tooth Type, n (%)				<0.001
Incisors	248 (22.1)	238 (32.9)	486 (26.3)	
Canines	168 (14.9)	144 (19.9)	312 (16.9)	
Premolars	284 (25.3)	144 (19.9)	428 (23.2)	
Molars	424 (37.7)	197 (27.2)	621 (33.6)	
Arch, n (%)				0.128
Maxilla	586 (52.1)	398 (55.0)	984 (53.3)	
Mandible	538 (47.9)	325 (45.0)	863 (46.7)	
Defect Depth (mm)				<0.001
Mean ± SD	5.8 ± 2.1	4.2 ± 1.6	5.2 ± 2.0	
Number of Walls, n (%)				-
1-wall	342 (30.4)	-	-	
2-wall	486 (43.2)	-	-	
3-wall	296 (26.3)	-	-	

**Table 2:** Performance metrics of machine learning models for defect classification

**Model**	**Accuracy (%)**	**Sensitivity (%)**	**Specificity (%)**	**Precision (%)**	**F1-Score**	**AUC-ROC (95% CI)**
**Deep Learning Models**						
3D-CNN	91.4	89.8	92.6	94.2	0.919	0.946 (0.928-0.964)
ResNet-18 3D	89.2	87.4	91.8	93.4	0.903	0.932 (0.912-0.952)
DenseNet-121 3D	88.6	86.8	90.4	92.6	0.896	0.924 (0.902-0.946)
**Traditional ML Models**						
Random Forest	86.8	84.6	89.2	91.2	0.878	0.912 (0.890-0.934)
Gradient Boosting	85.4	83.2	88.4	90.4	0.866	0.898 (0.874-0.922)
SVM (RBF)	83.6	81.4	86.2	88.6	0.848	0.876 (0.850-0.902)
Logistic Regression	78.2	75.8	81.4	84.2	0.798	0.824 (0.794-0.854)
K-Nearest Neighbors	76.4	73.6	79.8	82.4	0.778	0.802 (0.770-0.834)

**Table 3:** 3D-CNN model performance stratified by tooth type and defect characteristics

**Subgroup**	n	**Accuracy (%)**	**Sensitivity (%)**	**Specificity (%)**	**AUC-ROC**
**Tooth Type**					
Incisors	486	92.4	90.2	94.1	0.952
Canines	312	91.8	89.4	93.6	0.948
Premolars	428	93.2	91.6	94.8	0.958
Molars	621	88.6	86.8	90.4	0.926
**Arch Location**					
Maxilla	984	91.8	90.2	93.2	0.948
Mandible	863	90.8	89.2	92	0.942
**Defect Depth**					
<4 mm	524	89.2	86.8	91.4	0.928
4-6 mm	786	92.4	90.8	93.6	0.954
>6 mm	537	91.8	90.4	93.2	0.948
**Wall Configuration (Vertical)**					
1-wall	342	88.4	-	-	0.918
2-wall	486	92.6	-	-	0.956
3-wall	296	94.2	-	-	0.968
